# The Influence of Modified Silica Nanomaterials on Adult Stem Cell Culture

**DOI:** 10.3390/nano6060104

**Published:** 2016-06-04

**Authors:** Luigi Tarpani, Francesco Morena, Marta Gambucci, Giulia Zampini, Giuseppina Massaro, Chiara Argentati, Carla Emiliani, Sabata Martino, Loredana Latterini

**Affiliations:** 1 Department of Chemistry, Biology and Biotechnology, University of Perugia, Via Elce di Sotto, 8, 06123 Perugia, Italy; luigi.tarpani@unipg.it (L.T.); martygam@hotmail.it (M.G.); giulia.zampini87@gmail.com (G.Z.); pinamassaro9@gmail.com (G.M.); 2 Department of Chemistry, Biology and Biotechnology, University of Perugia, Via del Giochetto, 06123 Perugia, Italy; effemorena@gmail.com (F.M.); chiara.argentati89@gmail.com (C.A.); carla.emiliani@unipg.it (C.E.); sabata.martino@unipg.it (S.M.)

**Keywords:** silica nanoparticles, surface functionalization, adult stem cell, stem cell viability, cell morphology, fluorescence imaging

## Abstract

The preparation of tailored nanomaterials able to support cell growth and viability is mandatory for tissue engineering applications. In the present work, silica nanoparticles were prepared by a sol-gel procedure and were then functionalized by condensation of amino groups and by adsorption of silver nanoparticles. Transmission electron microscopy (TEM) imaging was used to establish the morphology and the average dimensions of about 130 nm, which were not affected by the functionalization. The three silica samples were deposited (1 mg/mL) on cover glasses, which were used as a substrate to culture adult human bone marrow-mesenchymal stem cells (hBM-MSCs) and human adipose-derived stem cells (hASCs). The good cell viability over the different silica surfaces was evaluated by monitoring the mitochondrial dehydrogenase activity. The analysis of the morphological parameters (aspect ratio, cell length, and nuclear shape Index) yielded information about the interactions of stem cells with the surface of three different nanoparticles. The data are discussed in terms of chemical properties of the surface of silica nanoparticles.

## 1. Introduction

A challenging objective in tissue engineering is the preparation of tailored nanomaterials able to support cell growth and viability [1,2]. Important research efforts are dedicated to the development of synthetic substrates able to ensure cell proliferation and able to provide biomimetic signals to cells in order to favor their development in an ordered network [3,4]. The cell morphology and activities (spreading, adhesion, migration, and proliferation) are highly sensitive to the chemical properties of the surfaces (composition, acidity, and hydrophobic/hydrophilic properties) and to the structure and porosity of the materials [5,6]. Due to their unique physico-chemical properties [7], nanostructured silica materials have found numerous applications in targeted drug delivery [8,9], theranostics [10,11], and biological labeling [12,13].

Silica nanoparticles can be prepared with a fine-tuning of the dimensions and morphology [7]. High versatility in the surface functionalization of silica nanomaterials can be achieved by simple condensation reactions of selected reactants, thus making it easy to modify the chemical nature of the silica surface.

A variety of ions or molecules can be entrapped within silica particles to control their release; different inorganic units can be attached or adsorbed on the silica matrix, resulting in new hybrid materials with added functional properties [14] that could give important contributions as templates for the driven growth and differentiation of stem cells.

To evaluate the potential applications of silica as nanomaterial for adult stem cell culture, we investigated the effect of differently functionalized silica nanoparticles, namely silica (SiO_2_), amine-functionalized silica (N-SiO_2_), and silver-silica hybrid (Ag-SiO_2_) nanoparticles, in terms of biocompatibility. We selected adult human bone marrow-mesenchymal stem cells (hBM-MSCs) and human adipose stem cells (hASCs) based on their ability to be easily isolated from adult tissues (bone marrow and lipoaspirate adipose tissues, respectively), expanded in culture, and induced to generate differentiated tissues [15,16,17,18,19].

We demonstrated that the surfaces of SiO_2_, N-SiO_2_, and Ag-SiO_2_ nanoparticles, respectively deposited on cover glasses, are suitable for hBM-MSCS and hASCs *in vitro* cultures and could represent a “functionalized substrate” for the delivery of active molecules to the cells.

## 2. Results and Discussion

### 2.1. Morphological Characterization of the Substrates

**Figure 1:**
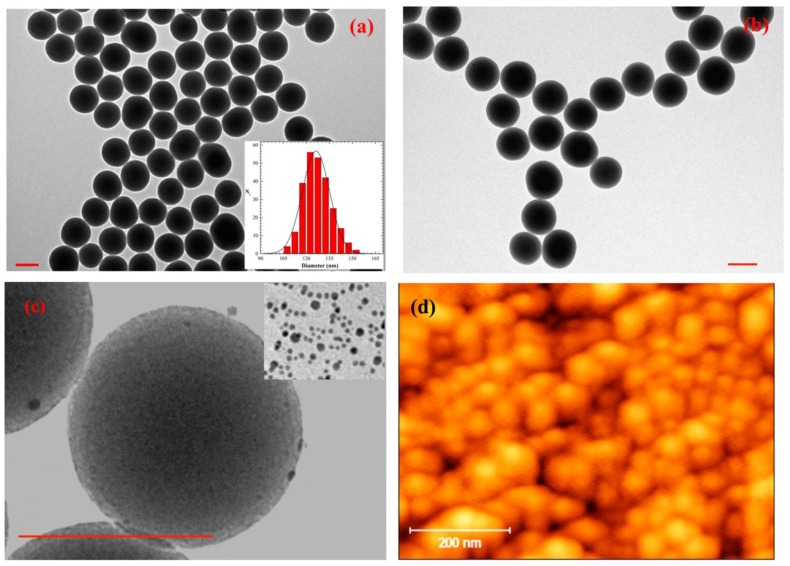
Transmission electron microscopy (TEM) images of SiO_2_ (**a**), inset size distribution histogram), N-SiO_2_ (**b**), and Ag-SiO_2_ (**c**), inset: TEM image of dodecanethiol-stabilized silver nanoparticles (DDT-Ag nanoparticles); (**d**) Atomic force microscopy (AFM) image of SiO_2_ nanoparticles deposited on cover glass after washing procedure. Scale bars are 200 nm.

Silica nanoparticles were prepared by a sol-gel procedure. The transmission electron microscopy (TEM) image reported in Figure 1a showed that SiO_2_ nanoparticles of spherical shape and with a quite narrow size distribution were formed. An average diameter of 126 ± 9 nm was determined from the statistical analysis of the TEM images (Figure 1a, inset). The amount of APTES used in the post-synthesis grafting step allowed the functionalization of the silica surface with amino groups (N-SiO_2_) without modifying the average dimension of the colloid. An example of a TEM image for sample N-SiO_2_ is shown in Figure 1b. Recently reported zeta potential values for similar SiO_2_ and N-SiO_2_ particles [20] indicated that the surface functionalization with amine groups confers a positive contribution to the surface charge distribution.

Silver nanoparticles of 5.3 ± 1.5 nm (inset Figure 1c for the TEM image) were successfully adsorbed on the amino-terminated silica nanoparticles as shown in Figure 1c (sample Ag-SiO_2_); the darker gains observed in Figure 1c could be assigned to silver colloids due to the larger cross section of silver atoms for electron scattering, compared to silicon. The efficient interaction of dodecanethiol-stabilized silver nanoparticles (DDT-Ag) with the silica colloid was promoted by the functionalization of the silica surface. The surface adsorption of DDT-Ag on silica is expected to confer a more hydrophobic character for the presence of alkyl chains in DDT. It has been demonstrated that the adsorption of surfactant-stabilized silver colloids on the silica surface does not dramatically impact the zeta potential values of the silica particles [21].

All the obtained silica particles were deposited on cover glasses. Atomic force microscopy (AFM) images recorded on the samples (see Figure 1d as an example) indicated that the amount of silica used (1 mg/mL) ensured a good particle density and a homogeneous distribution. It has to be noted that, in general, the particle coating produced a relatively smooth topography on the cover glasses, whose roughness appeared negligible compared to the cell dimensions. Thus, the supports did not present defined patterns or roughness to give preferential directions to the cell growth and adhesion.

### 2.2. Biological Characterization

#### 2.2.1. Stem Cell Viability on Surfaces of Differently Functionalized Silica Nanoparticles

To evaluate the cell viability, adult human bone marrow-mesenchymal stem cells (hBM-MSCs) and adult human adipose stem cells (hASCs), at a starting concentration of 1 × 10^3^ cells mL^−1^, were seeded on the surface of SiO_2_, N-SiO_2_, and Ag-SiO_2_ nanoparticles deposited on cover glasses according to respectively culture conditions. Measurements were performed at 3, 7, and 14 days after seeding.

**Figure 2:**
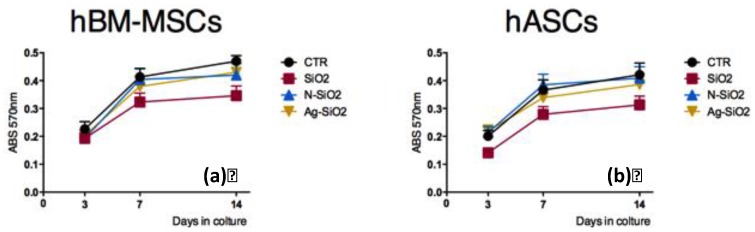
Stem cell viability on surfaces of SiO_2_, N-SiO_2_, and Ag-SiO_2_ nanoparticles: (**a**) (3-(4,5-Dimethylthiazol-2-yl)-2,5-Diphenyltetrazolium Bromide (MTT) viability assay of human bone marrow-mesenchymal stem cells (hBM-MSCs) on different nanoparticles at 3, 7, and 14 days; (**b**) MTT viability assay of hASCs on different nanoparticles at 3, 7, and 14 days.

We observed comparable trends of the mitochondrial dehydrogenase activity in hBM-MSCs cultured on the surface of SiO_2_, N-SiO_2_, and Ag-SiO_2_ nanoparticles deposited on glass coverslips, respectively, although, at each time point, each of these values were slightly lower than those on tissue cultured on polystyrene (CTR), as shown in Figure 2a. The lowest activity level was observed in hBM-MSCs cultured on the surface of SiO_2_ nanoparticles.

Similar results were obtained from the evaluation of viability of hASCs cultured on the surface of SiO_2_, N-SiO_2_, and Ag-SiO_2_ and on tissue culture polystyrene flasks (CTR), respectively (Figure 2b). Levels of activity were yet lower on SiO_2_, N-SiO_2_, and Ag-SiO_2_ cultures than tissue culture polystyrene flasks (CTR), with the lowest activity level in hASCs cultured on the surface of SiO_2_ nanoparticles (Figure 2b).

No signs of toxicity were observed in any of the hBM-MSC-nanoparticle or the hASC-nanoparticle cultures (data not shown).

The overall results indicate that the surfaces of SiO_2_, N-SiO_2_, and Ag-SiO_2_ nanoparticles are biocompatible substrates for human adult stem cell cultures. It has to be noted that, among the substrates tested in the present work, the SiO_2_ surface slightly slowed down the growth rate of both stem cell types. This observation might be related to the higher density of charges on the surface of bare silica.

#### 2.2.2. Interaction of Adult Stem Cells with Surfaces of Differently Functionalized Silica Nanoparticles

**Figure 3:**
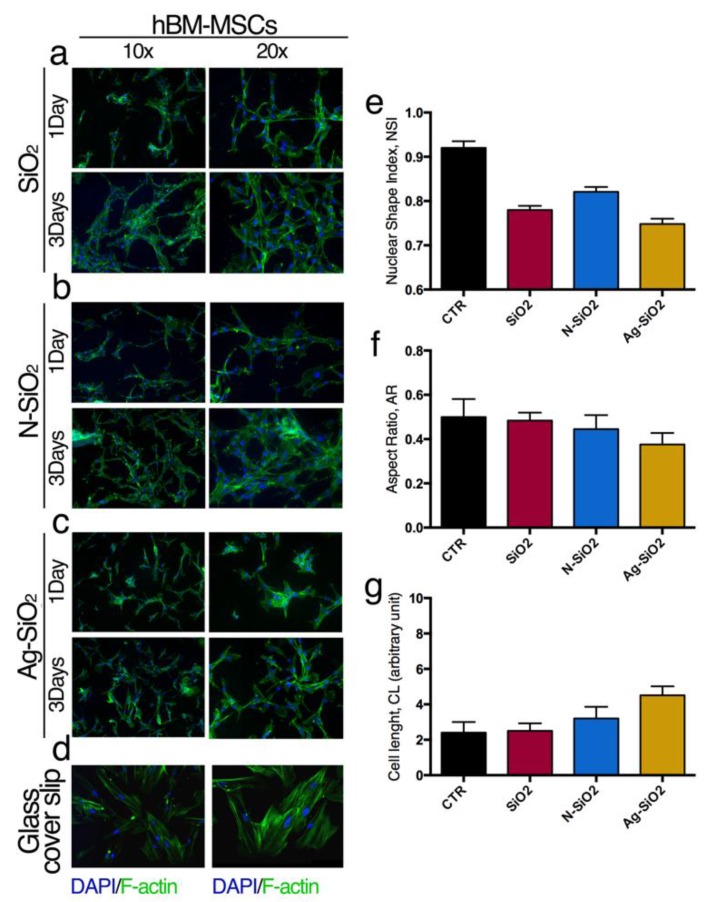
Interaction adult hBM-MSCs with nanoparticles: Immunofluorescence of hBM-MSCs seeded on (**a**) SiO_2_; (**b**) N-SiO_2_; (**c**) Ag-SiO_2_; (**d**) Control culture. Fluorescein isothiocyanate)-Phalloidin (FITC): F-actin; 4′,6-Diamidino-2-Phenylindole, Dihydrochloride (DAPI): nuclei. (**e**) Nuclear shape Index; (**f**) aspect ratio; (**g**) cell length. See method section for details.

**Figure 4:**
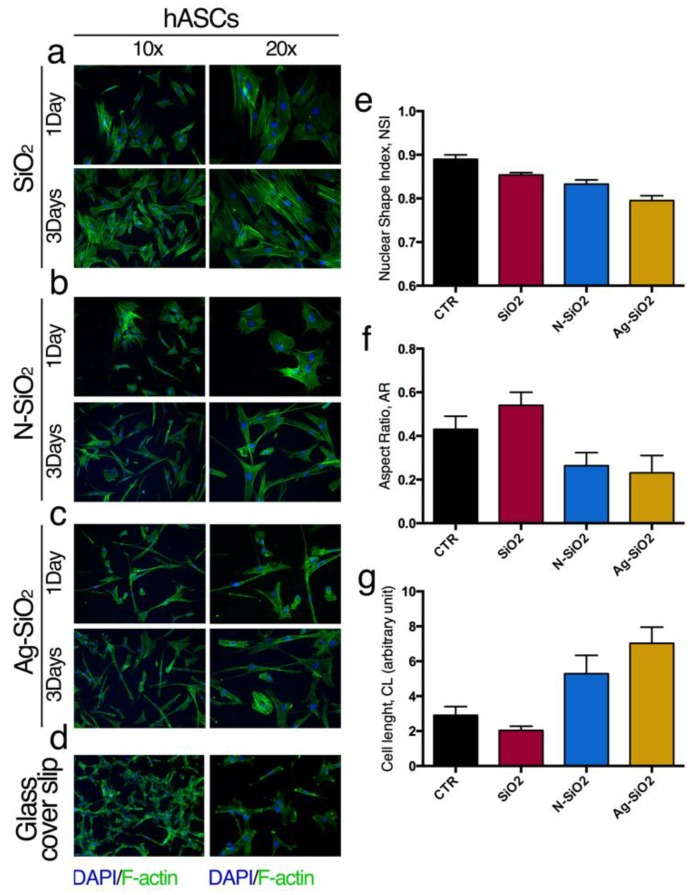
Interaction adult hASCs with nanoparticles: Immunofluorescence of hASCs seeded on (**a**) SiO_2_; (**b**) N-SiO_2_; (**c**) Ag-SiO_2_; (**d**) Control culture. FITC-phalloidin: F-actin; DAPI: nuclei. (**e**) Nuclear shape Index; (**f**) aspect ratio; (**g**) cell length. See method section for details.

The interactions of hBM-MSCs or hASCs with the surface of SiO_2_, N-SiO_2_, or Ag-SiO_2_ nanoparticles were evaluated analyzing the organization of cytoskeleton F-actin fibers (Figure 3 and Figure 4) and by measuring the aspect ratio (AR), cell length (CL) and nuclear shape index (NSI) of cells and nuclei, respectively (Figure 3 and Figure 4). These morphological data gave useful information about changes of the cell shape as a consequence of the interaction of the stem cells with the surface of nanoparticles.

Compared to the control cells seeded on glass coverslip, hBM-MSCs on the surface of SiO_2_ nanoparticles generated a network of stem cells, as revealed by the F-actin fluorescent staining (Figure 3a,d) and presented aligned and stretched nuclei (NSI, Figure 3e). No significant differences were observed in AR and CL cell parameters compared to the control reference, indicating that the whole cell morphology was not affected by the presence of the silica surface (Figure 3f,g). Interestingly, the cell network was evident after one day of seeding but it was maintained and became more intricate after three days in culture (Figure 3a).

HBM-MSCs on the surface of N-SiO_2_ samples showed a similar morphology of the stem cells on the surface of SiO_2_. In the former case, the immunofluorescence images showed a network of stem cells with a slightly elongated shape (Figure 3b,f,g) and nuclei aligned and stretched (Figure 3e). Again, the network was evident after one day of culture but became more convoluted after three days in culture (Figure 3b).

HBM-MSCs cultured on the surface of Ag-SiO_2_ samples were highly elongated and showed nuclei stretched with respect to the control stem cells cultured on a glass coverslip (Figure 3c,d,f,g). The cells grown on the surface of Ag-SiO_2_ nanoparticles also demonstrated a random distribution compared to the ones seeded on SiO_2_ and N-SiO_2_ samples (Figure 3).

F-actin architecture and cell morphological data revealed that hASCs have a larger shape when cultured on surfaces of SiO_2_ nanoparticles (Figure 4a,e–g) compared to the control cultures (Figure 4d).

These features were observed for hASCs cultured on N-SiO_2_ and Ag-SiO_2_ nanoparticles (Figure 4b,c). In fact, the cytoskeleton organization revealed a highly elongated cell morphology with random orientations (Figure 4b,c,f,g), and highly stretched nuclei (Figure 4e). These landscapes were evident after one and three days in culture (Figure 4) and were confirmed by measurements of NSI, AR, and CL parameters (Figure 4e–g).

These data suggest that the differences in the morphological parameters could be a consequence of the reduced charge density and to the higher hydrophobic character of the silica surfaces given the presence of amino-groups or DDT-Ag units, respectively, that could guide the cell elongation.

The overall results indicated that nanoparticles of SiO_2_, N-SiO_2_, and Ag-SiO_2_ were biocompatible with adult hBM-MSC and hASC cultures and they could be used as supports for biological applications. Further studies are necessary to elucidate the role of functional units in the establishment of specific interactions with cells.

## 3. Experimental Section

### 3.1. Materials

Tetraethylorthosilicate (TEOS, 98%), aqueous ammonia hydroxide (NH_4_OH, 28%–30%), 3-aminopropyltriethoxysilane (APTES, 99%), silver nitrate (AgNO_3_, 99.9+%), dodecanethiol (DDT, 98%), sodium borohydride (NaBH_4_, 98%), ethanol (EtOH, absolute, 99.8+%), and acetone (99.5+%) were all purchased from Sigma-Aldrich (Saint Louis, MO, US) and used without further purification.

### 3.2. Synthesis Procedures

#### 3.2.1. Preparation of Silica Colloids

Bare silica nanoparticles (SiO_2_) were synthesized using a base-catalysed sol-gel method. In a typical procedure, 2.6 mL of NH_4_OH and 0.5 ml of H_2_O were poured in ethanol (50 mL) followed by the dropwise addition of TEOS (1.1 mL) under stirring. The mixture was left to react for 24 h before recovering the nanoparticles by centrifugation at 3000 *g* for 15 min. The precipitate was washed twice with EtOH and then air-dried at room temperature. 

Amine-terminated SiO_2_ particles (N-SiO_2_) were prepared as follows: 0.18 mg of SiO_2_ were dissolved in EtOH (70 mL) followed by sonication for 15 min. A portion of 0.25 mL of APTES was then added to the solution, and the mixture was left under stirring for 16 h at room temperature. Afterwards, the solution was heated to reflux for 2 h and then cooled to room temperature. The nanoparticles were washed twice with EtOH (3000 *g*, 15 min) and dried. 

#### 3.2.2. Preparation of Ag Nanoparticles

An adaption of the method described by Kang *et al.* [22] was used to prepare DDT-stabilized silver nanoparticles. Briefly, 26.0 mg of AgNO_3_ were dissolved in 5 mL of ethanol in a water-ice bath followed by the addition of 25.0 μL of dodecanethiol. To this solution, ice-cooled NaBH_4_ (10 mL, 0.2 M) was added dropwise and kept under vigorous stirring for 10 min. The resulting silver colloidal suspension was left overnight at −18 °C in the freezer followed by centrifugation at 3000 *g* for 30 min. The precipitated nanoparticles were washed twice with ethanol, once with acetone, and finally solubilized in 15 mL of EtOH (sample DDT-Ag). 

#### 3.2.3. Preparation of the Ag/SiO_2_ Nanocomposite

In a 25 mL flask, 1.0 mL of sample DDT-Ag and 8.0 mL of N-SiO_2_ (1.0 mg/mL in EtOH) were mixed and left under stirring for 4 h to promote the adsorption of DDT-Ag on the silica surface. The resulting Ag/SiO_2_ nanocomposite was recovered by centrifugation (3000 *g*, 15 min), and the brown precipitate was washed twice with ethanol and once with acetone before drying (Ag-SiO_2_). 

#### 3.2.4. Characterization of the Colloids

A Philips 208 electron microscope (Philips, Amsterdam, The Netherlands) working at 80 kV was used to record the TEM images. A drop of the sample suspension in EtOH was deposited in a 300-mesh Formvar copper grid and allowed to dry in air overnight before the measurements. The size distribution of the colloids was determined by the statistical analysis of TEM images considering at least 250 nanoparticles for each sample. 

### 3.3. Biological Evaluation

#### 3.3.1. Isolation and Culture of Human Bone Marrow-Mesenchymal Stem Cells

Human bone marrow-mesenchymal stem cells (hBM-MSCs) were isolated and cultured as previously described [15,17].

Briefly, bone marrow cells were obtained from washouts of medullary cavities of femurs of informed patients undergoing primary total hip replacement. Bone marrow was diluted with phosphate buffer saline (PBS) without Ca^2+^/Mg^2+^ plus Ethylenediaminetetraacetic acid (EDTA). Mononuclear cells were isolated by density gradient on Lympholyte® (Cedarlane Laboratories Limited) and seeded in 25 cm^2^ culture flasks at a density of 2.5 × 10^6^ cells/mL in control medium consisting of RPMI-1640 (Euroclone) medium containing 10% heat-inactivated fetal bovine serum (FBS), 2 mM of L-glutamine, and 100 U mL^−1^ of penicillin-streptomycin (Euroclone) in a humidified atmosphere and 5% carbon dioxide (CO_2_) at 37 °C. After 5–7 days, the non-adherent cells were removed, and fresh medium was added to the flasks. After 15 days, a fibroblast-like colony started to grow. The medium was changed every three days. Cultured hBM-MSCs were analyzed by flow cytometry for their surface marker expression anti-CD44, -CD45, -CD73, -CD90, and -CD105 as already described [15].

#### 3.3.2. Isolation and Culture of Human Adipose-Derived Stem Cells from Lipoaspirate

Human adipose-derived stem cells (hASCs) were isolated and cultured as previously described [18].

Lipoaspirate adipose tissue, obtained by donor patients under written consent, according to an ethics committee, was crushed, extensively washed in PBS containing 5% penicillin/streptomycin (P/S) (EuroClone), and tissue fragments were incubated for 40 min at 37 °C, 5% CO_2_, with 0.075% collagenase Type I in PBS containing 2% P/S for tissue digestion and then neutralized with 5 mL of DMEM (Dulbecco’s Modified Eagle Medium; EuroClone) containing 20% FBS (EuroClone). The digested was centrifuged at 300 g, and the pellet was washed with PBS/2% P/S and centrifuged at 300 g for 5 min. Finally, the cell pellet was re-suspended in growth medium (DMEM supplemented with 20% FBS, 1% L-glutamine, 1% P/S) plated in tissue culture polystyrene (TCP) flasks and incubated at 37 °C, 5% CO_2_. hASCs started to grow as adherent fibroblast-like cells. The medium was changed every three days. 

#### 3.3.3. Culture of Stem Cells on the Surfaces of SiO_2_, N-SiO_2_, and Ag-SiO_2_ Nanoparticles

HBM-MSCs and hASCs were seeded on nanoparticle-coated glass coverslips in control medium. For the experiments, glass coverslips were sterilized through immersion in pure EtOH followed by three rinses in PBS, then deposited in a 24-well plate, and dried at room temperature. A portion of 100 μL of the re-suspended nanoparticles were coated on glass coverslips at a concentration of 1.0 mg/mL and dried over night under sterile conditions. The cover glasses were then rinsed with PBS solution. After that, 50 μL of hBM-MSC and hASC suspension (1 × 10^3^) in growth medium was plated on each substrate and incubated at 37 °C for 30 min for cell adhesion, and 500 μL of growth medium was then added. The medium was changed every three days. Experiments were carried out after 1, 3, 7, and 14 days of culture. As a control, similar experiments were performed seeding hBM-MSCs and hASCs on glass coverslips.

#### 3.3.4. Cell Viability Assay

To evaluate the cell viability, hBM-MSCs and hASCs were seeded on SiO_2_, N-SiO_2_, and Ag-SiO_2_ substrates at a starting concentration of 2 × 10^3^ cells mL^−1^ in control medium. As control, the same stem cell number was seeded on TCP. Cell viability was measured at different time points (3, 7, and 14 days in culture) by assaying the mitochondrial dehydrogenase activity through the MTT Formazan (M2003; Sigma-Aldrich, St Louis, MO, USA) assay for 4 h at 37 °C according to the manufacturer’s recommendations. The absorbance of the samples was measured using a microtiter plate reader (GDV) at 590 nm with a reference wavelength at 650 nm.

#### 3.3.5. Immunofluorescence

Immunofluorescence analyses were performed as previously described [15,16,17]. Briefly, cells were rinsed twice with PBS, fixed in 4% paraformaldehyde for 30 min, and, after PBS washing, cells were permeabilized, blocked (PBS + 10% FBS, 0.1% Triton X-100) for 1 h at room temperature (RT), and incubated with phalloidin (Alexa-fluor-488 phalloidin, Invitrogen, Grand Island, NY, USA) for 20 min. After being washed with PBS, samples were mounted, and nuclei were counterstained with Vectashield with DAPI (Vector Laboratories Inc., Burlingame, CA, USA). Images were acquired using fluorescence microscopy (Eclipse-TE2000-S, Nikon, Düsseldorf, Germany) using the F-ViewII FireWire camera (Soft Imaging System, Olympus, Münster, Germany)and elaborated by MATLAB (v.R2014b, MathWorks, Natick, MA, USA) and Fiji (Fiji Life-Line version, v.2015, U. S. National Institutes of Health, Bethesda, MD, USA).

#### 3.3.6. Cell and Nuclei Shape Analysis

To quantify the variation of nuclear shape index (NSI), the nuclei were measured by Fiji (Fiji Life-Line version, 22 December 2015) on fluorescent stained 4′,6-Diamidino-2-Phenylindole, Dihydrochloride (DAPI) images and used to calculate the NSI from the relationship: NSI = (4π × area)/(perimeter)^2^. NSI values range from 0 (elongated, elliptic morphology) to 1 (circular shape). 

Aspect ratio (AR), which is the ratio between the minor and major Feret diameter, is a measurement of cell elongation. AR was estimated by a custom-written MATLAB software (v.R2014b, MathWorks, Natick, MA, USA) that masked single cells from images of fluorescent-stained cells (Phalloidin) and loaded, thresholded with Fiji-implemented function, binarized, and analyzed for Feret’s diameter measurements. The AR assumes values between 0 (for spherical cells) and 1 (for cells with an elliptical shape with an axis ratio of 1:2).

The cell length (CL) values were calculated with a custom-written MATLAB software (v.R2014b) that masked single cells from images of fluorescent stained cells (Phalloidin) and traced the best-fitted outline ellipse to determine the major axis and the minor axis. Cell length was calculated from the relationship: CL = major axis/minor axis.

## 4. Conclusions

This work represents a proof-of-principle for generating surfaces of differently functionalized silica nanoparticles for stem cell culture. Silica nanoparticles were prepared by a basic-catalyzed sol-gel procedure (SiO_2_) and were then functionalized by condensation of amino groups (N-SiO_2_) and by adsorption of silver nanoparticles (Ag-SiO_2_). TEM imaging was used to establish the morphology and the average dimensions of about 130 nm, which were not affected by the functionalization. The three silica samples were deposited on cover glasses at a concentration value of 1 mg/mL, which produced a good particle density and a homogeneous distribution, as documented by AFM imaging. The differently coated cover glasses were used to culture hBM-MSCs and hASCs. The mitochondrial dehydrogenase activity determinations revealed comparable trends for hBM-MSCs and hASCs cultured on different nanoparticles surfaces, with respect to control culture conditions. Interestingly, we noted a small but constant slowdown of the proliferation rate for both hBM-MSCs and hASCs on the surface of SiO_2_ nanoparticles that, however, did not affect the stem cell survival and behavior. Through the analysis of immunofluorescence images, the morphological parameters of the cells were determined. These data worked to establish that a network of hBM-MSCs and a slightly elongated shape and aligned and stretched nuclei of hASCs were developed on N-SiO_2_ and Ag-SiO_2_ nanoparticles. This peculiar cell organization has been related to the physico-chemical properties of amino- and silver-functionalized surfaces.

Given that nanoparticles may be functionalized or loaded with different cargos, the procedure presented here could be useful for several biological applications including therapeutic applications, such as tissue engineering.
